# How to Grow Plump

**Published:** 1891-10

**Authors:** 


### HOW TO GROW PLUMP.

Any woman not predisposed to extreme thinness and in comparative good health can, by care to her diet and her temper, reach the state of plumpness she yearns for. But first of all she must not worry. Women who are nervous and worry over every trifle, will always remain thin. The young woman desiring to get fat must take life calmly; hurry about nothing; and as far as possible rest a great deal. If her nervousness is extreme, then she must apply to her physician, who will give her a sedative and a proper diet. She may eat freely of green salads, put upon them as much oil as she likes, but give up the vinegar.

